# Exploring the Functional Properties of Leaves of *Moringa oleifera* Lam. Cultivated in Sicily Using Precision Agriculture Technologies for Potential Use as a Food Ingredient

**DOI:** 10.3390/antiox14070799

**Published:** 2025-06-27

**Authors:** Carlo Greco, Graziella Serio, Enrico Viola, Marcella Barbera, Michele Massimo Mammano, Santo Orlando, Elena Franciosi, Salvatore Ciulla, Antonio Alfonzo, Rosario Schicchi, Daniela Piazzese, Carla Gentile, Luca Settanni, Giuseppe Mannino, Raimondo Gaglio

**Affiliations:** 1 Department of Agricultural, Food and Forest Sciences, University of Palermo, Viale delle Scienze, Bldg. 5, 90128 Palermo, Italy; carlo.greco@unipa.it (C.G.); enrico.viola01@unipa.it (E.V.); santo.orlando@unipa.it (S.O.); antonio.alfonzo@unipa.it (A.A.); luca.settanni@unipa.it (L.S.); raimondo.gaglio@unipa.it (R.G.); 2 Department of Biological, Chemical and Pharmaceutical Sciences and Technologies, University of Palermo, Viale delle Scienze, Bldg. 16, 90128 Palermo, Italy; graziella.serio01@unipa.it (G.S.); rosario.schicchi@unipa.it (R.S.); 3 Department of Earth and Marine Sciences, University of Palermo, Via Archirafi 22, 90123 Palermo, Italy; marcella.barbera@unipa.it (M.B.); daniela.piazzese@unipa.it (D.P.); 4 CREA—Research Centre for Plant Protection and Certification, Via Titina de Filippo 21, 90135 Palermo, Italy; massimo.mammano@crea.gov.it; 5 Research and Innovation Centre, Fondazione Edmund Mach (FEM), Via E. Mach 1, 38098 San Michele all’Adige, Italy; elena.franciosi@fmach.it; 6 Association of Producers SiciliaBio, Via Vittorio Emanuele 100, 92026 Favara, Italy; agrisinergie@gmail.com; 7 Innovation Centre, Department of Life Sciences and Systems Biology, University of Turin, Via Quarello 15/A, 10135 Turin, Italy; giuseppe.mannino@unito.it

**Keywords:** *Moringa oleifera* Lam. leaves powder, precision agriculture, solar smart dryer, microbiological quality, antiproliferative activity, cellular antioxidant activity

## Abstract

This study aimed to evaluate the microbiological quality and functional properties of *Moringa oleifera* Lam. leaves from plants cultivated in Sicily, with the objective of exploring their potential use in functional food production. Precision agriculture techniques, including unmanned aerial vehicle-based multispectral remote sensing, were used to determine the optimal harvesting time for *M. oleifera*. After harvesting, leaves were dried using a smart solar dryer system based on a wireless sensor network and milled with a laboratory centrifugal mill to produce powdered *M. oleifera* leaves (PMOLs). Plate counts showed no colonies of undesired microorganisms in PMOLs. The MiSeq Illumina analysis revealed that the class Alphaproteobacteria was dominant (83.20% of Relative Abundance) among bacterial groups found in PMOLs. The hydroalcoholic extract from PMOLs exhibited strong redox-active properties in solution assays and provided antioxidant protection in a cell-based lipid peroxidation model (CAA_50_: 5.42 μg/mL). Additionally, it showed antiproliferative activity against three human tumour epithelial cell lines (HepG2, Caco-2, and MCF-7), with GI_50_ values ranging from 121.03 to 237.75 μg/mL. The aromatic profile of PMOLs includes seven phytochemical groups: alcohols, aldehydes, ketones, esters, acids, terpenes, and hydrocarbons. The most representative compounds were terpenes (27.5%), ketones (25.3%), and alcohols (14.5%). Results suggest that PMOLs can serve as a natural additive for functional foods.

## 1. Introduction

Recently, the growing consumer demand for health-oriented foods has driven academic researchers and food producers to explore natural alternatives to enhance the functional value of processed foods [1]. This trend aligns with the principles of the National Recovery and Resilience Plan (NRRP), which aims to develop natural solutions that support both environmental sustainability and human health [2,3]. In this context, enriching foods with plant-derived products not typically used in culinary applications, such as leaves, peels, seeds, and skins, is a promising strategy to enhance their properties [4]. These products, depending on their botanical species of origin, are more or less rich in bioactive compounds that can prevent a wide range of chronic diseases [5,6,7], reduce oxidative stress, control inflammatory responses, and support immunological processes [8].

Among plant-derived products, *Moringa oleifera* Lam. leaves (MOLs) have gained particular attention for their antimicrobial, antioxidant, nutritional, and health-promoting properties [9]. *Moringa oleifera*, commonly known as the drumstick tree due to the appearance of its seed pods, or the horseradish tree because of the taste of its ground root preparations, is a deciduous shrub belonging to the Moringaceae family [10]. Its leaves are composite and alternate, capable of being bipinnate or partially tripinnate, with two to six pairs of opposite pinnate structures bearing leaflets in groups of three to five, while its elongated, linear pods turn brown when fully mature, its seeds are spherical and black, and its roots are white, swollen, and tuberous [11].

This plant has earned its reputation as a significant nutritional source and a reliable remedy within traditional medicine [12]. Numerous studies have examined the functional aspects of different organs of the plant, highlighting their high nutritional and phytochemical profiles [13]. In particular, MOLs contain higher levels of nutrients such as calcium, potassium, proteins, iron, vitamins, and minerals, as well as essential phytochemicals like alkaloids, anthraquinones, flavonoids, glucosinolates, phenolic acids, steroids, tannins, and terpenes, compared to other organs of the plant [14]. For this reason, they were classified by the World Health Organization (WHO) as a natural additive for the treatment of malnutrition [15].

The qualitative and quantitative composition of MOLs is influenced by various factors, including the cultivar, geographical location, soil type, climate, and other ecological conditions [16]. This variability is due to the remarkable adaptability of *M. oleifera* in diverse soils and climates [17]. Although native to tropical and subtropical regions of Africa, Asia, and Latin America [18], it has demonstrated the ability to thrive in areas where mild conditions have turned arid, such as the Mediterranean [19].

Sicily, located in the central Mediterranean basin, is characterized by smallholder farming, with crop production often dependent on rain-fed agriculture, especially in the inner rural areas [20]. These areas are affected by land abandonment [21], a situation worsened by climate change, which significantly impacts water availability [22]. Cultivating plants like *M. oleifera* could be a promising strategy to mitigate these issues, as it can help farmers diversify their income due to its adaptability to the climate changes affecting the region [17]. This plant could be integrated into the regional standard cropping systems to produce super forages for livestock feed [23] or used as a functional ingredient in health-focused foods.

To our knowledge, no studies regarding the microbiological and functional characterization of the leaves from *M. oleifera* grown in Sicily are present in the literature. Therefore, the specific objectives of this study are as follows: (i) assess the suitability of cultivating this climate-smart plant in Sicily, and (ii) evaluate the microbiological characteristics, phytochemical profile, antioxidant and anticancer activities, and volatilome composition of Sicilian powdered *M. oleifera* leaves (PMOLs) for their use in functional food production.

## 2. Materials and Methods

### 2.1. Experimental Site and Sustainable Cultivation Protocols

The study area is located in the countryside of Grotte, in the province of Agrigento (Italy). This part of Sicily is characterized by a hot-summer Mediterranean climate [24] and a soil moisture regime that is xeric, bordering on arid, with a thermic temperature regime. Specifically, *M. oleifera* plants were cultivated at the “Morreale” farm (37°22′52.284″ N, 13°40′24.067″ E, 380 m a.s.l), which specializes in the cultivation of medicinal and aromatic plants (MAPs) and is recognised for its environmentally sustainable and multifunctional operations that contribute to soil protection in Sicily [25,26].

The experimental field for *M. oleifera* contains 300 three-year-old plants of the Indian genus, produced and multiplied by the Morreale nursery farm. The plants were spaced 150 cm apart in rows, with uniform spacing of 200 cm between rows. The plant density was 30,000 plants per hectare, and this plot is being used to verify the feasibility of mechanical harvesting of *Moringa* [27]. During the vegetative period (March–November), inter-row surface tillage was periodically performed to control weed growth and disrupt soil capillarity. No organic or mineral fertilization was applied during the growth period of *M. oleifera*.

### 2.2. Moringa oleifera Precision Farming

The use of unmanned aerial vehicles (UAVs) equipped with multispectral cameras and post-processing software has become a prevalent method for determining vegetation indices (VIs) in the management of MAPs. This approach aims to modernize agricultural practices by reducing resource consumption and enhancing productivity [28]. Precision aromatic crops (PACs) are increasingly gaining scientific attention, with successful implementation relying on various technologies such as sensors for crop monitoring, global navigation satellite systems (GNSSs), variable rate application (VRA) machinery, geographic information systems (GISs), and decision support systems (DSSs) [28].

These technologies enable spatially variable crop condition monitoring. Sensors like multispectral cameras, which capture limited spectral bands, play a crucial role in evaluating crop health. Spectral data help identify vegetation status, which is vital for precise crop management. This study focuses on determining the optimal harvest time for *M. oleifera* using UAVs equipped with multispectral sensors. The UAV, a DJI Mavic 3 Multispectral drone, (Dà-Jiāng Innovations Science and Technology Co., Ltd., Shenzhen, China), was used to capture multispectral images with five CMOS sensors that cover visible and near-infrared bands, including green, red, RedEdge, and NIR. These sensors provide high-resolution images, with the camera system integrated into the UAV platform to autonomously fly with predetermined routes.

The flight mission involved the careful planning of parameters using DJI Pilot 2 software (version 2.5), which included height, speed, direction, and camera settings, such as shot sequence and image overlap. The flights took place under favourable conditions, with clear skies and low wind, following a predefined route and waypoints.

After capturing the multispectral images, they were pre-processed to generate a multiband ortho-mosaic using Agisoft Metashape Professional (version 1.7.3). This photogrammetric software produces 3D and 2D spatial data for GIS applications [28]. The process involved constructing a Dense Cloud, followed by creating the Mesh and digital elevation model. The final steps involved orthorectification and mosaicking to generate the multiband ortho-mosaic. Spectral canopy data were then extracted from the ortho-mosaic using QGIS (version 3.16.6 Hannover) and analysed through an object-based image analysis approach. The multispectral data were processed with the open-source software QGIS (version 3.16.6 Hannover), applying Geographic Object-Based Image Analysis and calculating normalized difference vegetation index (NDVI) values to assess vegetation health [28].

The NDVI, calculated using the formula

NDVI = ((NIR − R))/((NIR + R)),(1)

was used to separate canopy areas from soil surfaces.

**Figure 1:**
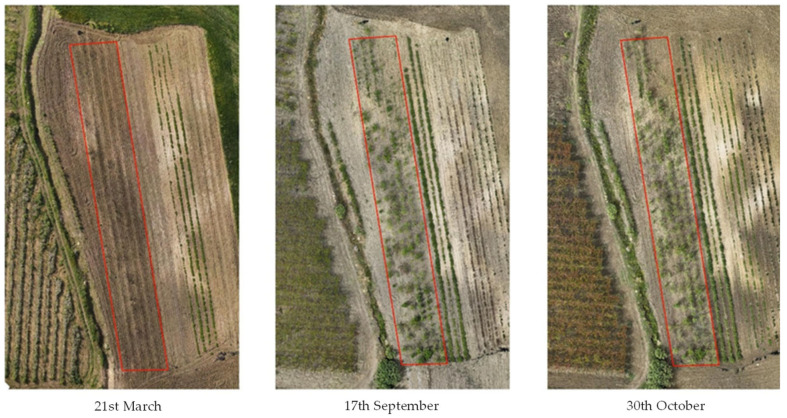
*Moringa oleifera* monitoring through precision agriculture.

The collected biomass was intended for the drying process, so it was necessary to identify the time of highest vegetative vigour for *M. oleifera*. The NDVI is sensitive to crop biophysical properties like nitrogen, chlorophyll, vigour, and biomass. To choose the optimal harvest time, *M. oleifera* was monitored year-round, and NDVI values were calculated from March to November 2024. *Moringa oleifera* red, green, and blue (RGB) images (Figure 1) from March 21, September 17, and October 30, 2024, show different vegetative vigour.

### 2.3. Moringa oleifera Leaves Collection, Drying, and Powder Production

*Moringa oleifera* leaves were randomly harvested from plants using stainless steel dissecting scissors and placed in sterile plastic bags. The plant was identified by Prof. Rosario Schicchi, who also deposited a specimen in the Herbarium Mediterraneum Panormitanum (PAL) of the Botanical Garden of the University of Palermo, under the voucher number 109787. The leaves were promptly transferred to Morreale farm, where they were washed following a modified version of the procedure described by Miceli et al. [29]. This involved a 30 min wash in a 0.2% (*v*/*v*) chlorinated water solution, followed by a rinse with tap water to remove any residual chlorine. The washed leaves were first spun dry using a manual salad spinner and then dried with trays using a smart solar dryer system based on a wireless sensor network (WSN) [25,26]. Sampling was carried out in triplicate over three consecutive weeks, from the end of October to mid-November 2024.

The drying process utilized forced hot-air convection to remove moisture from the fresh herb biomass (82.8 kg), maintaining a temperature of 40 °C and 25% humidity to reduce moisture while preserving quality. Each 48 h drying cycle extracted about 208 L of water. A dehumidification machine with a capacity of 52.8 kg per 24 h, along with a reversible compression heat pump, maintained the drying chamber temperature between 10 °C and 30 °C. The dryer was powered by electricity from photovoltaic panels on the structure, generating 8383 kWh annually. Each drying cycle consumed 190 kWh of energy [30,31].

The dried leaves were then transported in a portable refrigerator to the Laboratory of Agricultural, Food, and Environmental Microbiology (University of Palermo). There, they were milled using a Retsch centrifugal apparatus (Mill ZM1, Haan, Germany) equipped with a 250 μm stainless steel ring sieve, and the resulting PMOLs was stored at 4 °C until further analysis.

### 2.4. Safety Evaluation by Culture-Dependent Approach

The microbiological assessment of PMOLs was conducted using a culture-dependent approach, following the methodology described by Chulibert et al. [32]. Briefly, ten grams of powder were homogenized in 90 mL of Ringer’s solution (Sigma-Aldrich, Milan, Italy), serially diluted in the same diluent, and placed on selective agar media used to enumerate the undesired populations generally contaminating food matrices. The different microbial groups were inoculated, cultivated, and incubated as follows: total mesophilic microorganisms (TMMs) on plate count agar (PCA), incubated at 30 °C for 72 h; Enterobacteriaceae family members on violet red bile glucose agar (VRBGA), incubated at 37 °C for 24 h; *Escherichia coli* on *E. coli*-coliforms chromogenic medium (CHROM), incubated at 37 °C for 24 h; coagulase-positive staphylococci (CPS) on Baird Parker (BP) agar with rabbit plasma fibrinogen (RPF) supplement, incubated at 37 °C for 48 h; pseudomonads on Pseudomonas agar base (PAB) supplemented with cephaloridine sodium fusidate cetrimide (CFC), incubated at 25 °C for 48 h; and yeasts and moulds on dicloran rose-bengal chloramphenicol (DRBC) agar, incubated at 25 °C for 48 h and 7 days, respectively. The detection of *Listeria monocytogenes* and *Salmonella* spp. was carried out on 25 g of PMOLs, according to ISO 11290-1 [33] and ISO 6579-1 [34] standards, respectively. The analyses were performed in triplicate and all media were purchased from Oxoid (Milan, Italy).

### 2.5. Culture-Independent Analysis of Bacterial Community

Total genomic DNA was extracted from PMOLs using the Microbiome DNA Isolation Kit (Norgen Biotek Corp., Thorold, ON, Canada), according to the manufacturer’s instructions. The extracted DNA was then used to amplify the bacterial V3-V4 variable region of the 16S rRNA gene, as reported by Busetta et al. [35]. To evaluate the amplicon library and remove primer dimers, a Bioanalyzer 2100 (Agilent, Palo Alto, CA, USA) with a High Sensitivity DNA Kit (Agilent) was used. The cleaned amplicons were then pooled in equimolar ratios and subjected to paired-end sequencing on the Illumina MiSeq system (Illumina, San Diego, CA, USA) at Fondazione Edmund Mach (FEM, San Michele a/Adige, Italy). The resulting sequences were quality-checked using the DADA2 workflow [36]. Taxonomic analyses for bacteria were performed using the Greengenes 13_8 99% database for Operational Taxonomic Units (OTUs). The FASTQ-formatted sequences were deposited in the NCBI sequence read archive (SRA) under the project ID PRJNA1246356.

### 2.6. Preparation of the Powdered Moringa oleifera Leaves Extract

The hydroalcoholic extract of PMOLs was prepared by individually extracting three samples (3 g each) with 70% ethanol. Each sample was extracted three times with 30 mL of solvent per cycle (extraction ratio of 1:10, g/mL), for a total of 90 mL of solvent per sample. This procedure was adopted to ensure maximum compound recovery. The resulting extracts were centrifuged (10 min at 10,000× *g*, 4 °C) and filtered through a Millex HV 0.45 μm filter (Millipore, Billerica, MA, USA). The supernatants were then collected, combined, and concentrated under reduced pressure using a rotary evaporator to obtain a final extract concentration of 55 mg/mL. This concentrated extract was used for the determination of total polyphenolic content and antioxidant properties. Additionally, the extract was employed to assess the bioactivity of PMOLs on human cancer epithelial cells.

### 2.7. Content of Pigments and Polyphenol Compounds

The total polyphenolic content (TPC) of PMOLs was quantified in the hydroalcoholic extract of PMOLs using the Folin–Ciocalteu colorimetric assay, based on the reduction in the Folin–Ciocalteu reagent in an alkaline environment, resulting in the formation of a blue pigment. The intensity of the blue pigment was measured at 735 nm [37,38]. Gallic acid (GA) calibration curves were used for quantification, and results were expressed as milligrams of gallic acid equivalents (GAEs) per 100 g of dry weight (DW). All assays were performed in triplicate to ensure precision and reliability.

The chlorophyll and carotenoid contents in PMOLs were determined according to the method described by Lichtenthaler and Buschmann [39], with minor modifications described by Gugliuzza et al. [40]. Briefly, 5 g of PMOLs was mixed with 5 mL of dimethyl sulfoxide and incubated at 65 °C for 6 h. After incubation, the mixture was centrifuged at 15,000× *g* for 5 min. The supernatant was collected, and absorbance readings were taken at 664 nm for chlorophyll *A* (Cha), 648 nm for chlorophyll *B* (Chb), and 470 nm for carotenoids. The concentrations of chlorophylls and carotenoids were calculated using the following equations:

Chlorophyll *A* (Cha) = 12.25 × A664 − 2.79 × A648 (2)

Chlorophyll *B* (Chb) = 21.50 × A648 − 5.10 × A664 (3)

Carotenoids = (1000 × A470 − 1.82 × Cha − 95.15 × Chb)/225(4)

### 2.8. Cellular Culture

The human epithelial cell lines Caco-2 (colon cancer), MCF-7 (breast cancer), and HepG2 (hepatocarcinoma) were sourced from the American Type Culture Collection (Rockville, MD, USA). Caco-2 and MCF-7 cells were maintained in DMEM, while HepG2 cells were cultured in RPMI medium (VWR International, Radnor, PA, USA). Both media were enriched with 5% FBS (VWR International, Radnor, PA, USA), 2 mM L-glutamine (VWR International, Radnor, PA, USA), 50 IU/mL penicillin, and 50 μg/mL streptomycin (VWR International, Radnor, PA, USA). Cells were grown in a controlled environment at 37 °C with 5% CO_2_ [41]. Subcultures were performed in 75 cm^2^ flasks, and cells were detached using trypsin-EDTA (VWR International, Radnor, PA, USA) when they reached approximately 80% confluence.

### 2.9. Antioxidant Activity

The antioxidant properties of the hydroalcoholic extract of PMOLs were evaluated using a combination of classical in-solution assays and a cell-based lipid peroxidation model.

#### 2.9.1. Antioxidants Properties

The in-solution assays included the ABTS^•+^ and DPPH^•^ assays to assess radical scavenging activity, and the FRAP assay to measure metal-reducing antioxidant potential [42].

For the ABTS^•+^ assay, the ABTS^•+^ radical was generated by incubating 7 mM ABTS with 2.45 mM K_2_S_2_O_8_ at room temperature overnight. The resulting ABTS^•+^ solution was diluted in methanol to achieve an absorbance of 0.70 at 734 nm. Radical decay, induced by the incubation with different extract dilutions, was monitored by measuring the decrease in absorbance at 734 nm.

In the DPPH^•^ assay, the DPPH^•^ radical, dissolved in 95% (*v*/*v*) ethanol, was mixed with various dilutions of the PMOLs extract. The mixture was incubated for 40 min at room temperature in the dark. The reduction in the DPPH^•^ radical was measured by monitoring absorbance at 517 nm.

For the FRAP assay, the reaction mixture, consisting of 300 mM CH_3_COONa (pH = 3.6, HCl), 10 mM TPTZ, and 20 mM FeCl_3_ in an 8:1:1 (*v*/*v*/*v*) ratio, was added to the diluted sample. The mixture was incubated at 37 °C for 30 min, and absorbance was recorded at 600 nm.

Calibration curves for all assays were constructed using hydroxy-2,5,7,8-tetramethylchroman-2-carboxylic acid (Trolox) as a standard antioxidant, and the results were expressed as mmol Trolox Equivalents (TEs) per 100 g of dry weight (DW).

#### 2.9.2. Cellular Antioxidant Activity

The cellular antioxidant activity (CAA) assay was performed following the method outlined by Wolfe and Rui [43], with slight modifications described by Mannino et al. [44]. In brief, HepG2 cells were seeded in 96-well plates at a density of 6.0 × 10^4^ cells/well in complete culture medium and incubated for 24 h. Afterward, the cells were treated with 25 μM DCFH-DA (Sigma Aldrich, St. Louis, MO, USA) and varying concentrations of the PMOLs hydroalcoholic extract for 2 h. The final ethanol concentration did not exceed 0.25% (*v*/*v*). Control and blank wells received 25 μM DCFH-DA in culture medium containing 0.25% ethanol (*v*/*v*). Following the incubation, cells were washed with PBS (Sigma Aldrich, St. Louis, MO, USA), and 600 μM 2,2′-Azobis(2-methylpropionamidine) dihydrochloride (ABAP) (Sigma Aldrich, St. Louis, MO, USA) in Hanks’ Balanced Salt Solution (HBSS) (Sigma Aldrich, St. Louis, MO, USA) was added to the treated and control wells (positive control). Blank wells (negative control) were treated with HBSS alone. Fluorescence in treated, control, and blank wells was measured every 5 min over the course of 1 h using a plate reader at 37 °C. The area under the fluorescence versus time curve was integrated to determine the CAA values for treated and control wells using the following equation:

CAA = 100 × [100 − (∫SA/∫CA)](5)

where ∫SA represents the integrated area of the sample wells, and ∫CA represents the integrated area of the control wells. The antioxidant activity was quantified as CAA_50_, which is the concentration of WRSP extract required to inhibit 50% of DCF formation. CAA_50_ was derived from a concentration–response curve (CAA) using linear regression analysis and was expressed as mg of dry weight (DW) per mL of cell medium. The final result, presented as mg/mL of cell medium, is the average of three independent experiments.

### 2.10. Antiproliferative Activity

The 3-(4,5-Dimethyl-2-thiazolyl)-2,5-diphenyl-2H-tetrazolium bromide (MTT) assay was used for evaluating the antiproliferative activity of the PMOLs extract on exponentially growing cells, as previously described by Mannino et al. [38]. Briefly, cells were seeded in standard 96-well plates at appropriate cellular densities for each cell line. Preliminary growth curve analyses were performed to determine the optimal seeding density that would ensure the cells remained in the exponential phase of growth throughout the entire duration of the treatment. After 24 h of incubation, the PMOLs extract was added at concentrations ranging from 500 to 50 μg DW/mL in the cell culture medium, and the cells were further incubated for an additional 48 h. The ethanol concentration in the wells did not exceed 0.25% (*v*/*v*). Control wells contained cells incubated with culture medium plus 0.25% ethanol (*v*/*v*). Following incubation, 0.5 mg/mL MTT reagent (Sigma Aldrich, St. Louis, MO, USA) was added to each well, and after 3 h at 37 °C, the reagent was removed. The blue formazan crystals produced by viable cells were solubilized in dimethyl sulfoxide (DMSO), and absorbance was measured at 570 nm using a microplate reader (UV-1900i, Shimadzu^®^, Milan, Italy). The percentage of cell growth (PG) relative to the control (untreated cells) was calculated using the following equation:

PG = 100 × (ODtest − ODtzero)/(ODctr − ODtzero)(6)

where ODtest represents the average optical density after cell exposure to the extract for a specific period of time, ODtzero is the average optical density before extract addition, and ODctr is the average optical density after the incubation period with untreated cells. The concentration required to achieve 50% growth inhibition (GI_50_) for each cell line was determined from the concentration–response (PG) curves using linear regression analysis. Results are expressed as mg of dry weight (DW) per mL of cell medium. The final values are the mean of three independent experiments.

### 2.11. HPLC-DAD-MS/MS Analysis

Chromatographic and mass spectrometric analyses were carried out using a high-performance liquid chromatography (HPLC) system from Agilent Technologies (Model 1200, Santa Clara, CA, USA), which was integrated with a diode array detector (DAD) and interfaced to an LC-MS setup employing the Agilent 6330 Series Ion Trap system. The mass spectrometer was equipped with an electrospray ionization (ESI) source, enabling the sensitive detection of polar compounds. The separation of the analytes was achieved through reverse-phase chromatography on a Luna C18 column (3 μm particle size, 150 mm × 3.0 mm internal diameter; Phenomenex, Torrance, CA, USA). The mobile phase was delivered at a constant flow rate of 0.2 mL per min, and the column temperature was precisely controlled at 25 °C using an Agilent 1100 Series G1316A thermostatted column compartment. Solvent A was composed of Milli-Q-grade water acidified with 0.1% (*v*/*v*) formic acid, while Solvent B consisted of acetonitrile, also acidified with 0.1% (*v*/*v*) formic acid. The chromatographic run began with an initial solvent ratio of 90% A and 10% B, which was maintained for the first 5 min. This was followed by a linear gradient in which the proportion of Solvent B was gradually increased to 55% over the next 25 min. The gradient was then extended to reach a final composition of 70% of Solvent B by 50 min. Upon completion of the gradient, the mobile phase was returned to the original 90:10 ratio (A:B) and held for an additional 10 min to allow for column re-equilibration before the next sample injection. A sample volume of 10 µL was injected for each analysis. UV-Visible spectral data were collected across a wavelength range of 220 to 650 nm, with chromatograms specifically monitored at 280 nm (phenolic acids) and 360 nm (flavonols). The identification and quantification of individual flavonoids were further supported by tandem mass spectrometry (MS/MS) analyses performed in negative ionization mode, following previously established protocols [45].

### 2.12. Volatile Organic Compounds Analysis

The volatile organic compounds (VOCs) in PMOLs were analysed using solid-phase microextraction (SPME) combined with gas chromatography–mass spectrometry (GC–MS). A DVB/CAR/PDMS SPME fibre (50 mm, Supelco, Bellefonte, PA, USA) was exposed to 5 g of the sample while continuously stirring at 60 °C for 5 min. After extraction, the fibre was inserted into a GC splitless injector, where VOCs were desorbed for 1 min at 250 °C. Chromatographic separation was performed using a DB-624 capillary column (Agilent Technologies, 60 m, 0.25 mm, 1.40 µm). The oven temperature program started with a 5 min isotherm at 40 °C, followed by a linear increase of 5 °C/min up to 200 °C, which was maintained for 2 min. Helium was used as the carrier gas at a flow rate of 1 mL/min. The interface temperature was maintained at 230 °C, and mass spectra were recorded in full-scan mode across a range of *m*/*z* 40–400 amu. Individual VOCs were identified by comparing their mass spectra with those in the NIST05 commercial library. Prior to each analysis, a procedural blank was run by exposing the fibre to the same extraction conditions in the absence of sample, in order to check for potential background contamination; no interfering signals were detected.

Identified compounds were reported by normalizing GC-MS peak areas with the total area of selected peaks. Results, derived from triplicate analyses, were expressed as relative percentages of significant peaks.

### 2.13. Statistical Analysis

A one-way ANOVA analysis was conducted, followed by Tukey’s multiple comparison test with a significance level of *p* < 0.05 for pairwise comparisons of antiproliferative activity results. Statistical analyses were performed using SPSS Statistics 24 software (IBM Corp., Chicago, IL, USA).

## 3. Results and Discussion

### 3.1. Moringa oleifera Optimal Harvest Time

**Figure 2:**
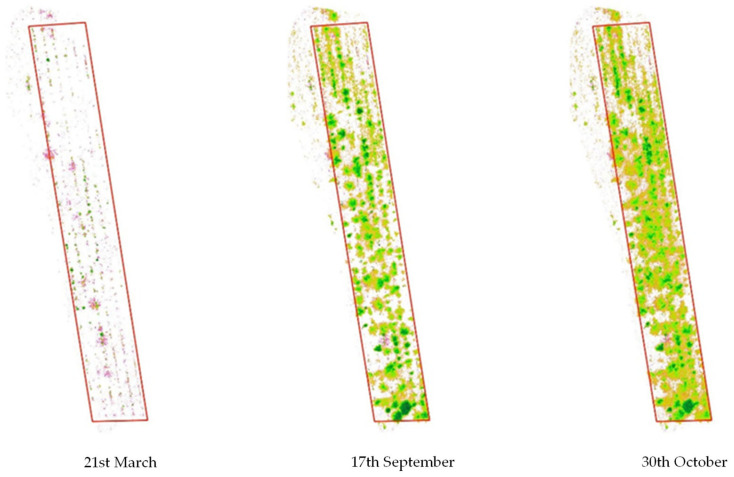
*Moringa oleifera* NDVI values during vegetative period.

NDVI values were calculated during three main periods: March, September, and October, to identify areas of vegetation. Zonal statistics in QGIS facilitated the calculation of surface areas corresponding to different NDVI classes, enabling precise management. The spectral data also enabled the creation of false-colour images, which highlighted vegetation and provided insights into the health and vigour of *M. oleifera*. Typically, NDVI values ranged from 0.7 to 0.9, indicating robust growth. On 21st March, the average NDVI values were 0.3, 89% of the land surface was bare, and *M. oleifera* plants were lacking vegetation. On 17th September, more than half of the surface (54%) showed healthy vegetation with good vigour [46]. By 30th October, 67% of the surface showed a good level of vegetative vigour (Figure 2).

**Figure 3:**
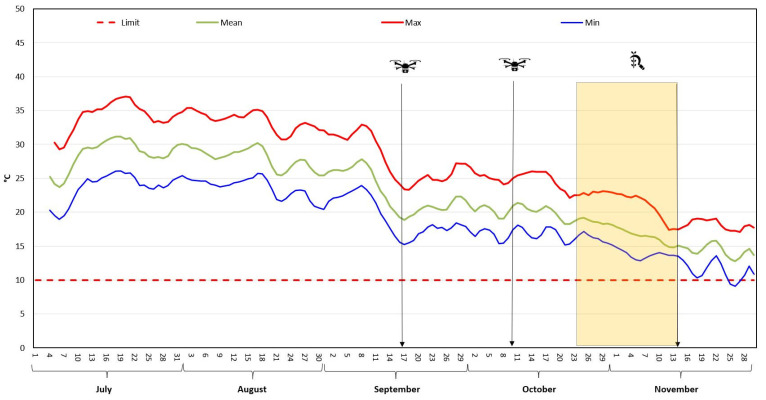
Temperature profile and *Moringa oleifera* harvesting time NDVI value and mean min temperature.

Overall, UAV-based multispectral imaging, coupled with advanced data processing tools, provides valuable insights for optimizing crop management and decision-making in precision farming, particularly for MAPs like *M. oleifera* [47]. The optimal harvest time was determined when mean NDVI values were 0.85 and the mean minimum temperature was above 10 °C, the threshold below which *M. oleifera* plants begin to lose their leaves and experience a decrease in vegetative vigour [48]. This period was between 30th October and 14th November 2024 (Figure 3).

This framework was compared with multiple other species to evaluate cross-species consistency in harvest indicators.

For instance, pomegranate (*Punica granatum* ‘Wonderful’) demonstrates an optimal harvest period from October 12 to 27, defined by various fruit quality metrics including juice percentage, soluble solids, and antioxidant levels [49]. With regard to Durum wheat, phenological NDVI sampling at the first node stage has been applied to determine sampling time, although temperature data are not specified [50]. Forest types in Shennongjia, Central China, rely on mean temperatures during maturity stability phases (T_popt_, i.e., temperature during the period of physiological maturity), which range from 22.72 °C to 24.05 °C and correspond to periods of peak NDVI values [51].

Indian pennywort (*Centella asiatica*) achieves an optimal biomass and centelloside production at 25 °C, while a significantly reduced growth is observed at 15 °C [52].

The NDVI remains a widely utilized metric across studies for assessing vegetation health and guiding harvest decisions, as evident in *Moringa* and other crops [50,53,54]. Temperature similarly emerges as a key environmental factor, with species-specific thermal optima reinforcing the significance of thermal thresholds in harvest timing [50].

### 3.2. Drying Curves

**Figure 4:**
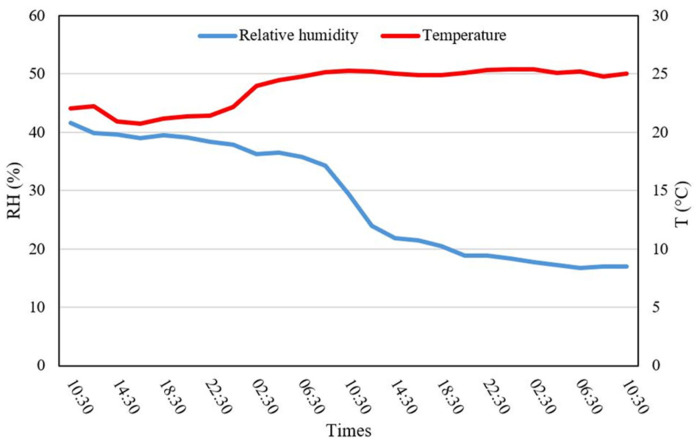
Drying chamber profiles in two cycle days.

The drying process began at 10:30 a.m. and concluded after two days, during which the relative humidity dropped from 41 to 9%. Temperature fluctuations were monitored, and the data were transmitted via Wi-Fi to a ThingSpeak account for real-time tracking. The WSN system enabled the precise monitoring of the moisture content and drying rates for *M. oleifera*, providing valuable decision support throughout the drying process (Figure 4).

In summary, the solar-powered drying system efficiently removed moisture while adhering to renewable energy guidelines, ensuring a sustainable approach to drying aromatic crops like *M. oleifera*. At the end of the drying cycle, the moisture content was measured at 9% for *M. oleifera*, similar to findings by Moyo et al. [55], who employed an air-drying method under shaded conditions, and Tetteh et al. [56], who employed various drying techniques. The fresh-to-dry weight ratio was 100/42, with the final weight of the dried biomass being 34.8 kg.

The drying of *M. oleifera* using sun, shade, and cabinet methods revealed that sun drying provided the highest antioxidant capacity and mineral retention [57]. An indirect solar drying system effectively reduced the moisture content of *Moringa* leaves by 25% within four hours, outperforming conventional open sun drying in speed and efficiency [58]. In comparison, a solar hot-air drying system decreased drying time for turnips by 10 h, compared to traditional approaches [59]. For alfalfa, advanced systems such as the double-pass solar air collector (DPSAC) and photovoltaic thermal collector (PVT) achieved thermal efficiencies of 73.7% and a coefficient of performance (COP) of 12.5, respectively [60].

Indirect solar drying also demonstrated superior preservation of leaf colour in red amaranth and coriander compared to open sun drying, suggesting better nutrient conservation [58]. The integration of phase change materials (PCMs) in solar dryers further enhanced the quality of dried agricultural products by maintaining stable drying temperatures and minimizing nutrient degradation [61]. The indirect solar drying system for *Moringa* exhibited faster drying rates and improved preservation of both colour and nutrients compared to open sun drying [58]. More broadly, solar drying systems incorporating PCMs and hybrid configurations displayed high thermal efficiencies and shortened drying durations.

The economic analysis of solar drying systems revealed favourable outcomes. The indirect solar dryer used for *M. oleifera* had a short payback period of 0.78 years [58], and direct solar drying systems demonstrated increased economic efficiency across a range of crops [62].

### 3.3. Microbial Count

The PMOLs produced in this study underwent plate counts to assess the presence of the main undesired populations associated with foods [63]. This investigation is crucial because their origin exposes them to environmental contamination from water, soil, and air, making them potential carriers of spoilage and pathogenic microorganisms [64]. Therefore, members of the Enterobacteriaceae family, CPS, and *E. coli*, which serve as process hygiene indicators [65], along with *Salmonella* spp. and *L. monocytogenes*, associated with food safety criteria [66], were investigated in PMOLs.

PMOLs did not host detectable levels of the main food pathogenic bacteria, which are known to cause foodborne illness outbreaks globally [67]. No colonies of pseudomonads, yeasts, or moulds responsible for food spoilage [68] were detected in the PMOLs under investigation. Only TMMs showed the presence of viable cells, with levels of 2.55 ± 0.33 CFU/g. In addition to being classified as environmental contaminants [69], these microorganisms were found at densities that do not pose a health risk, as they were well below the maximum limit of 4.0 Log CFU/g set by the International Commission on Microbiological Specifications for dried vegetables [70]. The positive effect of the drying and sanitation process was further confirmed when comparing the values of the present study with a study in which fresh cut salads and sprout samples were microbiologically characterised. As Seow et al. [71] indeed demonstrated, higher levels of aerobic mesophilic counts (approximately 6.5 Log CFU/g), total coliforms (approximately 5.5 CFU/g), and the presence of presumptive *Salmonella* spp. colonies in 15% of lettuce samples were reported. These findings indicated that the drying and thermal process could be crucial in enhancing the safety of this kind of product. In contrast, when vegetable products, such as cabbage slices, carrot slices, or several fruits were dried, a significant reduction in the concentration of various alterative or pathogenic microorganisms was observed [72]. The exceptional hygiene and safety standards of the PMOLs produced in this study can be attributed to the good practices applied during the harvesting and sanitation of the leaves, along with the thermal treatment applied during the drying process [73]. These results underscore the microbiological suitability of Sicilian PMOLs as a natural additive in novel food production.

### 3.4. Composition of Bacterial Communities

**Figure 5:**
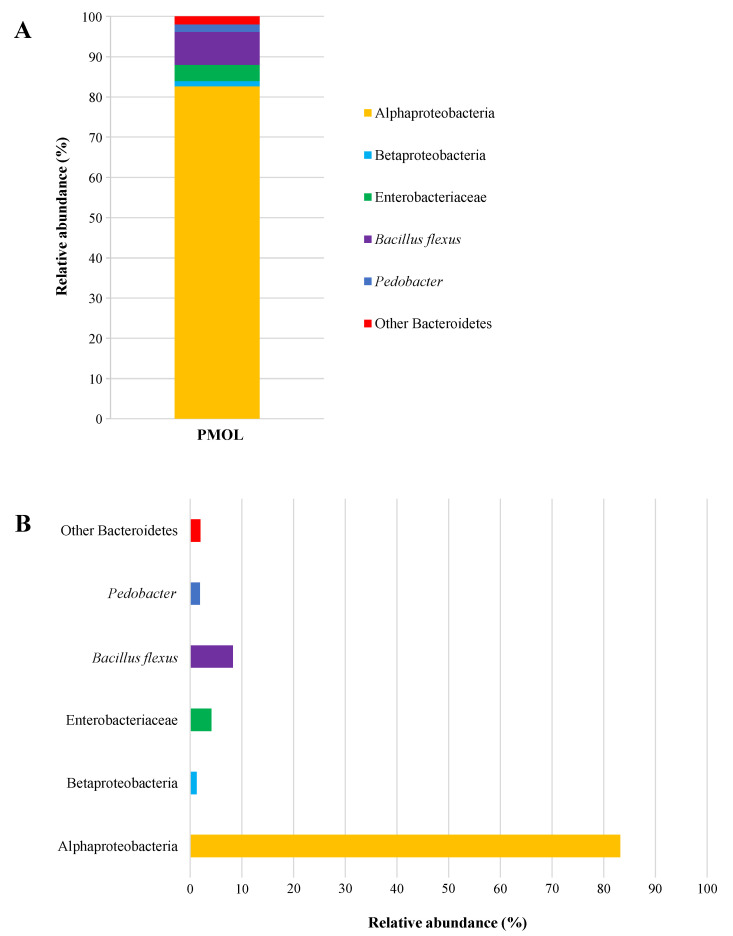
Relative abundances (%) of bacterial community identified by Illumina technology. (**A**): total bacterial composition; (**B**): single taxonomic groups. Abbreviation: PMOLs, powdered *Moringa oleifera* leaves.

In this study, DNA-based Illumina technology was employed to thoroughly examine the microbiota of PMOLs. This culture-independent microbiological approach allows for the evaluation of the complete bacterial composition in both processed and unprocessed foods [74], including the identification of viable but non-cultivable and/or dormant bacterial communities [75]. The results reported in Figure 5A include the OTUs with a Relative Abundance RA ≥ 0.1%, which are classified as abundant bacterial communities [76]. In particular, six taxonomic groups were identified at RA ≥ 0.1%, divided into one phylum, two classes, one family, one genus, and one species (Figure 5B).

The class Alphaproteobacteria was the dominant bacterial group found in PMOLs, accounting for 83.20% of the RA, and their presence is imputable to environmental contamination [77]. However, this presence is not surprising, as similar values were previously reported by Garofalo et al. [78] in commercially available *M. oleifera* leaf powder. The species *Bacillus flexus*, which has received the “Qualified Presumption of Safety” status from the European Food Safety Authority [79], was detected in the analysed sample with an RA of 8.24%. Members of the Enterobacteriaceae family were found with an RA of 4.07%, while bacteria from the phylum Bacteroidetes (Bacteroidota), the class Betaproteobacteria, and the genus *Pedobacter* each had an abundance of approximately 1.5%. These microorganisms are soil-associated bacteria [80,81], and their presence in PMOLs is due to cross-contamination with soil [82]. Notably, the four primary foodborne pathogens (*E. coli*, CPS, *Salmonella* spp., and *L. monocytogenes*) were absent in PMOLs, corroborating the findings from the culture-dependent approach.

### 3.5. Content of Pigments and Polyphenol Compounds in Powdered Moringa oleifera Leaves 

The phytochemical characterisation of the hydroalcoholic extract of PMOLs focused on the major bioactive compounds present in the leaves, with particular emphasis on the total polyphenol content (TPC). Polyphenols are widely recognised for their biological relevance and are often responsible for the bioactive properties of plant extracts [44]. Reported TPC values for *Moringa* leaf samples vary widely, ranging from a few milligrams to about twenty grams per 100 g of dry product. This variability is largely influenced by the geographical origin and pedoclimatic conditions, which affect the phytochemical profile both qualitatively and quantitatively [83,84]. The estimated TPC value for our sample is consistent with that reported in the literature for *Moringa* leaves from different geographical areas. This suggests that *Moringa* plants grown in Sicily could also represent a valuable nutraceutical resource. In addition to environmental factors, several studies have highlighted the crucial role of extraction conditions. In particular, the polarity of the extraction mixture has a significant impact on the yield of polyphenols. The polarity of a solvent mixture can be estimated using empirical values, such as the polarity index ET (30) and the dielectric constant εr. Because the polarity of mixed solvents does not vary linearly with their proportions, it can be approximated by interpolation based on the polarity values of pure solvents. In a study by Rocchetti et al. [83], the extraction of polyphenols was investigated using three different solvent mixtures: 100% methanol, methanol–water (50:50), and ethyl acetate. The highest polyphenol yield was obtained using methanol, with comparable results between the 100% methanol and methanol–water mixtures. On the other hand, Pollini et al. [84] analysed the effect of extraction parameters—including temperature, extraction time, solid/liquid ratio, and methanol percentage—and concluded that the polarity of the mixture was the most influential factor. Under all conditions, significantly higher extraction yields were observed with methanol–water (50:50) than with pure methanol. In our study we used an ethanol–water (70:30) mixture for extraction. Based on the dielectric constant as an empirical measure of polarity for methanol, water and ethanol, we estimate that our ethanol–water (70:30) mixture has a polarity similar to methanol–water (50:50), but higher than pure methanol. This suggests that the chosen hydroalcoholic mixture has a strong extraction capacity, at least with respect to the phenolic fraction.

Although the estimated TPC value for our sample (2.86 g GAE/100 g DW) is lower than that determined for Tunisian *Moringa* samples extracted in butanol (10 g/100 g DW) [85], methanolic extracts of Egyptian *Moringa* samples (15 g GAE/100 g DW) [86], or an Angolan *Moringa* sample extracted in a hydroalcoholic mixture at a high temperature (26.7 g GAE/100 g DW) [87], it is in line with or exceeds the values reported for *Moringa* samples from different geographical origins extracted in hydroalcoholic mixtures at room temperature (18.6 mg GAE/100 g − 1.08 g GAE/100 g DW) [84,86,88,89].

In addition, the content of carotenoids and chlorophylls in our sample of Sicilian PMOLs was determined by a colorimetric method described by Lichtenthaler and Buschmann [39]. The levels of these pigments in plant tissues can serve as valuable indicators of the ideal growth conditions for plants. Moreover, these pigments are not only important for the photosynthetic efficiency of the plant, but also reflect the adaptation of the plant to its environment in view of their role in protecting the plant against oxidative stress, especially under stressful environmental conditions.

On the other hand, from a health perspective, the presence of carotenoids and chlorophylls in the plant matrix enhances their functional value, particularly in the context of fortified foods and nutraceutical applications. In addition to their well-known antioxidant role, carotenoids support immune function, visual health, and skin protection. Chlorophyll has also been associated with several beneficial effects, including detoxification, anti-inflammatory properties, and the promotion of overall cellular health [90].

The total carotenoid content of PMOLs was determined to be 1.64 mg per 100 g dry weight, expressed as the beta-carotene equivalent (BBE). Although this carotenoid content is relatively modest—especially when compared to other plant sources such as carrots, spinach, mangoes, and apricots, as well as certain widely consumed spices [91], it is in line with the typical carotenoid content found in the leaves of many plants.

In addition to carotenoids, we determined the content of chlorophyll *a* and chlorophyll *b*, which are the main pigments involved in photosynthesis and overall plant metabolism. The concentration of chlorophyll *a* in our leaf sample was 1.01 g/100 g, while chlorophyll *b* was 0.34 g/100 g, resulting in a total chlorophyll content of 1.35 g/100 g and a chlorophyll *a*/*b* ratio of 2.97, which is typical for green plants [40].

The chlorophyll *a* and chlorophyll *b* levels and their ratio, as well as the carotenoid content observed in the Sicilian PMOLs, indicate an efficient light-harvesting system under the local growing conditions. This indicates that *Moringa* plants are well adapted to the pedoclimatic conditions of Sicily. When grown under optimal environmental conditions, such as mild temperatures and the abundant sunlight typical of the region, *Moringa* seems to be able to thrive while maintaining high levels of bioactive compounds. These results highlight the resilience of *Moringa* in non-native environments and suggest that its cultivation in temperate zones may be a viable and promising option.

### 3.6. Phytochemical Composition

Data analysed on PMOLs revealed a complex and well-represented polyphenolic composition, with particularly high concentrations of phenolic acids and glycosylated flavonoids. Among the most abundant compounds is quinic acid, with 125.5 mg per 100 g of DW, followed by caffeic acid derivatives such as 3-caffeoylquinic acid (101.3 mg/100 g) and 4-caffeoylquinic acid (36.2 mg/100 g) (Table 1).

These compounds, known as precursors of chlorogenic acids, have been extensively studied for their antioxidant and anti-inflammatory properties and their ability to modulate glucose metabolism [92]. Their abundance confirms what has already been observed in the literature regarding the health-promoting potential of *M. oleifera*, especially when subjected to gentle preservation processes such as freeze-drying, which is more effective than thermal drying in preserving thermolabile molecules. The flavonoid content is also noteworthy (Table 1). The data show a predominance of glycosylated forms over free aglycones, as often observed in other phytochemical studies as well. Quercetin 3-*O*-glucoside (70.1 mg/100 g) and isorhamnetin 3-*O*-glucoside (69.3 mg/100 g) appeared to be among the most represented flavonoids, followed by kaempferol 3-*O*-rutinoside (50.6 mg/100 g) and other derivatives such as kaempferol 3-*O*-glucoside (33.1 mg/100 g). The relevant presence of these glycosylated forms is particularly important, since these molecules are generally more stable and potentially more bioavailable than aglycones, such as quercetin (9.3 mg/100 g) or kaempferol (11.3 mg/100 g), which are present in significantly lower concentrations [93].

Another interesting aspect is the presence of flavones such as vicenin-2 (26.2 mg/100 g) and apigenin 8-C-glucoside (48.6 mg/100 g), less common compounds but also known for their biological activities, including neuroprotective effects and immune system modulation.

The combined presence of these different classes of polyphenols suggests a bioactive synergy that could contribute to the beneficial effects observed in in vitro and in vivo studies on *M.oleifera*.

Many of the identified compounds, including quinic acid, chlorogenic acid, quercetin, and kaempferol, as well as their glycosylated forms (e.g., vicenin-2, quercetin-3-*O*-glucoside, kaempferol-3-*O*-glucoside, and quercetin-3-*O*-rutinoside), have already been reported in the leaves, seeds, flowers, and other parts of *M. oleifera*. The occurrence of rhamnose-containing flavonoid glycosides, such as rutin and vicenin-2, is also in agreement with earlier reports, which describe *M. oleifera* as a plant rich in flavonoids and glycosylated derivatives, potentially contributing to the enhanced stability and bioavailability of these molecules [94,95,96].

While our study confirms the presence of these known phytochemicals, the quantitative profile and the relative abundance of several glycosylated flavonoids may vary based on environmental conditions, cultivation site, and extraction protocol. To our knowledge, few studies have provided detailed quantitative data at this level of resolution, especially for Sicilian *M. oleifera*, and this could contribute to explaining some of the biological activities we observed.

### 3.7. Antioxidant Properties

Plant matrices are rich sources of bioactive molecules with redox-active properties, particularly polyphenolic derivatives. Extensive evidence indicates that such molecules play a key role in modulating cellular redox homeostasis, thereby mitigating oxidative stress. This protective action occurs through the direct scavenging of reactive species, modulation of antioxidant enzyme activity, and in some cases, upregulation of related gene expression.

The phytochemical profiling of PMOLs revealed a high content of polyphenolic compounds, particularly phenolic acids and flavonoids. Notably, many of the identified flavonoids are present in glycosylated forms, which are generally more stable and potentially more bioavailable than their aglycone counterparts. This structural feature may enhance their ability to exert redox-active effects within cells, thus contributing to biologically relevant antioxidant activity.

The antioxidant capacity of extracts from Sicilian PMOLs was evaluated by a combination of in vitro assays, including both solution-phase analyses, where reactive species were generated in a hydrophilic reaction medium, and a lipid peroxidation model using intact cells. This integrative approach allowed the characterisation of both the redox-active properties of the phytochemicals present in the sample, which may contribute to the intracellular pool of soluble antioxidants, and their ability to permeate biological membranes and exert protective effects within the cellular milieu. The quantitative parameters describing the antioxidative properties of PMOLs are presented in Table 2.

The results obtained indicate that PMOLs have significant radical scavenging and metal-reducing properties, as demonstrated by their reducing ability in solution-based assays. This is reflected in the FRAP value of 15.71 ± 2.19 mmol TE per 100 g DW, which is slightly lower than the values reported for methanol and methanol–water extracts of Italian *M. oleifera* leaves (23.27 and 21.67 mmol TE per 100 g DW, respectively), but substantially higher than that of ethyl acetate extracts (5.50 mmol TE per 100 g DW) [83]. The ABTS^•+^ value of 8.03 ± 0.49 mmol TE per 100 g FW is also within the lower range compared to methanol (14.85 mmol TE/100 g DW) and methanol–water extracts (18.08 mmol TE/100 g DW), yet higher than the ethyl acetate extract (3.05 mmol TE/100 g DW) [68]. Similarly, the DPPH^•^ value of 5.67 ± 0.49 mmol TE per 100 g DW is lower than methanol (18.13 mmol TE/100 g DW) and methanol–water extracts (19.71 mmol TE/100 g DW) but higher than ethyl acetate (9.40 mmol TE/100 g DW) [83].

Although these comparisons are useful to contextualize the antioxidant potential of Sicilian *M. oleifera* leaves, it is important to underline that a direct comparison of the literature data can be challenging due to the variability in extraction protocols, solvent polarities, and expression units adopted across studies. Differences in sample preparation, such as drying techniques, extraction temperatures, or solvent ratios, can significantly affect the yield and profile of antioxidant compounds.

Nonetheless, our results also align with those obtained for *Moringa* leaves from other geographical regions. For instance, our DPPH^•^ value of 5.67 mmol TE per 100 g DW is slightly lower than the 9.31 mmol TE per 100 g DW reported by Younis et al. [97] for *Moringa* leaves from Pakistan, but still demonstrates a considerable antioxidant activity.

Beyond the chemical assays, the biological relevance of the extract’s antioxidant potential was confirmed by the CAA assay, which revealed a CAA_50_ value of 5.42 ± 0.43 µg DW per mL cell medium. This result highlights the intracellular protective effects of *M. oleifera*’s bioactive properties, demonstrating their ability to permeate cell membranes and mitigate oxidative stress at physiologically relevant concentrations. Given the well-documented association between dietary antioxidants and the reduced risk of oxidative stress-related disorders, these findings support the potential of Sicilian *M. oleifera* leaves as a valuable dietary source of natural antioxidants.

### 3.8. Antiproliferative Activity

*Moringa oleifera* has been extensively studied for its bioactive properties, particularly its anticancer potential. Different organs of the plant, including leaves, seeds, bark, and roots, have demonstrated cytotoxic effects against various cancer cell lines. However, most research has focused on the anticancer activity of *M. oleifera* leaf extracts due to their rich phytochemical composition.

Several studies have reported the cytotoxic and pro-apoptotic effects of *M. oleifera* leaf extracts in both in vitro and in vivo models. Hydroalcoholic extracts of *M. oleifera* leaves have shown selective cytotoxicity towards leukaemic cells, with an IC_50_ value of 150 µg/mL, while sparing normal peripheral blood mononuclear cells (PBMCs). This effect was associated with cell cycle arrest, mediated by increased levels of the cyclin-dependent kinase inhibitor p21 [98]. In further support of this cytotoxic activity, Parvathy and Umamaheshwari [99] reported that *M. oleifera* leaf extracts exerted both cytotoxic and pro-apoptotic effects on lymphoid cells. Recent findings by Potestà et al. [100] confirmed these results, demonstrating cytotoxicity against both lymphoid and monocytic cancer cells, while maintaining selectivity for tumour cells. No toxicity was observed in normal PBMCs [100].

The selective cytotoxic potential of *M. oleifera* extracts extends to melanoma cells, as shown by their inhibitory effects on A375 and A2058 human melanoma cells, in contrast to normal human skin fibroblasts (WS1) and primary normal human dermal fibroblasts (NHDFs). This activity was associated with increased mitochondrial ROS production and elevated levels of caspase-3 and -7, indicating apoptosis induction [101].

In addition, the anticancer effects of *M. oleifera* leaves have been demonstrated in epithelial tumour cell lines, including oesophageal, lung, liver, and cervical cancer cells. An aqueous leaf extract exhibited cytotoxic and pro-apoptotic effects on oesophageal cancer cells, with an IC_50_ value of 389.2 µg/mL at 24 h [102]. Similarly, in A549 lung cancer cells, *M. oleifera* extracts induced oxidative stress through decreased Nrf2 levels and increased expression of p53 and caspases-3, -7, and -9 [103]. Similar effects were observed in HepG2 hepatocellular carcinoma cells, where Jung et al. [104] showed that a water-soluble extract induced apoptosis, causing changes in DNA content and cell cycle arrest. In HeLa cervical cancer cells, the extracts exhibited antiproliferative activity through G0/G1 cell cycle arrest, increased caspase-3 levels, and the upregulation of the cell cycle inhibitor p27 [105]. These findings were further corroborated by studies showing an IC_50_ value of 70 µg/mL for *M. oleifera* leaf extracts in HeLa cells, coupled with apoptosis induction [105].

In our study, we evaluated the cytotoxic potential of the hydroalcoholic extract from PMOLs in three epithelial cancer cell lines: HepG2 (hepatocellular carcinoma), Caco-2 (colorectal adenocarcinoma), and MCF-7 (breast cancer). Our findings (Table 3) revealed differential sensitivity among these cancer cell lines, with MCF-7 cells exhibiting the highest sensitivity (GI_50_: 121.03 ± 3.71 µg/mL), followed by Caco-2 cells (GI_50_: 182.36 ± 4.52 µg/mL), while HepG2 cells showed the highest resistance (GI_50_: 237.75 ± 4.37 µg/mL). These results suggest that the efficacy of PMOLs extracts may be cell-type-dependent, possibly influenced by differences in metabolic activity, redox homeostasis, and specific molecular targets in each cancer type.

A previous study by Charoensin [103] similarly evaluated the cytotoxic activity of *M. oleifera* leaf extracts on HepG2, Caco-2, and MCF-7 cell lines using methanolic and dichloromethane extracts. Despite the higher antioxidant capacity of the methanolic extract, the dichloromethane extract showed greater antiproliferative activity, with IC_50_ values ranging from 112 to 133 μg/mL, compared with 250 μg/mL for the methanolic extract. Our results fall within this range, with comparable values for MCF-7 cells but slightly higher GI_50_ values for Caco-2 and HepG2, which may reflect differences in extraction methods, phytochemical composition, or experimental conditions [106].

Although different experimental conditions may contribute to explain the observed differences in GI_50_ values compared to the literature data, it cannot be excluded that a unique phytochemical profile of the *M. oleifera* leaf extract used in our study, both in terms of qualitative composition and quantitative abundance of bioactive compounds, could justify the observed cytotoxic activity. Although different experimental conditions may contribute to the variability in GI_50_ values compared to th eliterature data, it cannot be excluded that the unique phytochemical profile of the *M. oleifera* leaf extract used in our study—both in terms of the qualitative composition and quantitative abundance of bioactive constituents—may underlie the observed antiproliferative effects. In particular, several compounds identified in our extract, including kaempferol, quercetin, and chlorogenic acid, have been extensively reported in the literature to exert antiproliferative activity in vitro in models of colorectal, breast, and liver cancer [107,108,109,110,111,112]. This suggests that the cytotoxic effects observed in Caco-2, HepG2, and MCF-7 cell lines may be at least partially attributed to the presence of these polyphenolic constituents.

Despite the effects on breast and liver cancer, cells may have in vivo significance only if the bioavailability of the extract’s phytochemicals in the bloodstream is demonstrated, and the high sensitivity of Caco-2 colorectal cancer cells to the extract suggests a potential protective role of *M. oleifera* consumption in colorectal cancer prevention. Indeed, a local effect on intestinal epithelial cells does not necessarily depend on systemic bioavailability, and the GI_50_ value we determined for Caco-2 cells (182.36 ± 4.52 µg/mL) falls within a range that could realistically be achieved in the intestine following the dietary intake of *M. oleifera* leaves. These findings support further investigations into the potential role of *M. oleifera* as a functional food for the prevention of colorectal cancer.

Taken together, these studies confirm the potent and selective cytotoxic effects of *M. oleifera* leaf extracts on cancer cell types and further support the potential of Sicilian PMOLs as promising candidates for anticancer research and therapeutic applications.

### 3.9. Volatilome Composition

**Figure 6:**
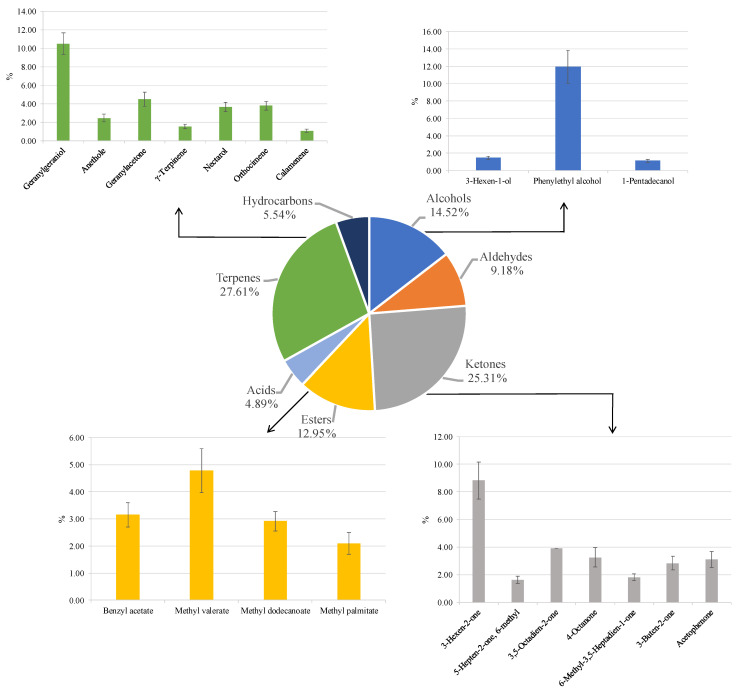
Volatile organic compounds emitted from powdered *Moringa oleifera* leaves. Results indicate mean percentage values ± standard deviation (S.D.) of three measurements and are expressed as relative peak areas (peak area of each compound/total area of the significant peaks to all samples) × l00.

The VOC profiles emitted from PMOLs were analysed using the solid-phase microextraction gas chromatography–mass spectrometry (SPME-GC–MS). This technique identified 25 volatile compounds across seven phytochemical groups: alcohols, aldehydes, ketones, esters, acids, terpenes, and hydrocarbons. The most prevalent classes were terpenes (27.6%) and ketones (25.3%), followed by alcohols (14.5%) and esters (12.9%). Acids (4.9%), hydrocarbons (5.5%), and aldehydes (9.2%) were present in smaller amounts (Figure 6).

Phenylethyl alcohol (11.9%), known for its floral aroma [113], was the primary representative of the alcohol class. Ketones were notably abundant, with 3-Hexen-2-one (8.8%) being the predominant compound, recognised for its roasted flavour and chestnut-like scent [114]. This compound has also been identified as the most abundant ketone in PMOLs in previous studies [115]. Esters, which significantly influence aroma and odour profiles, particularly contributing to fruity and floral nuances [116], were represented by methyl valerate (4.8%).

Terpenes were the most abundant and significant group. These compounds, primarily produced by plants, some insects, and yeast [117], are known for their floral and green odours [118]. Geranylgeraniol was the most abundant terpene identified, with similar findings reported in *Moringa* flowers cultivated in Nigeria [119]. Geranylgeraniol is recognised for its broad spectrum of biological activities, including antimicrobial, therapeutic, anti-inflammatory, and antioxidant effects [120,121,122,123]. Another notable terpene, geranyl acetone, was also identified in *Moringa* flowers [119]. This compound is characterized by its green, fruity, waxy, woody, pear, rosy, and guava-like aromas with tropical notes, and it is recognised for its broad bioactivity, including germicidal and antimicrobial properties [124].

Overall, the aromatic profile identified in this study is rich and complex. Some of the compounds detected have been previously reported in *M. oleifera* [115,119,125,126,127]. However, only a limited number of studies have investigated the volatile composition of PMOLs, and the data vary significantly depending on the geographical origin of the samples [118].

## 4. Conclusions

The results of this study indicate that *M. oleifera* exhibits a high degree of adaptability to the pedoclimatic conditions typical of the Sicilian region. The use of the NDVI allowed for the determination of the optimal harvest time, while the implementation of a smart solar drying system enabled the production of PMOLs with high microbiological quality standards. In particular, chemical analyses of PMOLs revealed a complex and well-represented polyphenolic composition, particularly rich in phenolic acids and glycosylated flavonoids, which are generally more stable and bioavailable than their aglycone counterparts. The thermal drying process employed proved effective in preserving thermolabile molecules, resulting in phenolic content values higher than those reported in previous studies using conventional drying methods. From a functional standpoint, the resulting PMOLs product demonstrated strong radical scavenging and antioxidant activity, along with moderate anticancer effects. The analysis of the aromatic profile shows that, although the product was used in its dried form, it retained a rich aromatic composition, including ketones, alcohols, and terpenes, as well as bioactive compounds such as geranylgeraniol and geranyl acetone. These findings suggest that Sicilian PMOLs could be incorporated into innovative food products to enhance their functional properties.

## Figures and Tables

**Table 1:** Polyphenolic compounds detected in powdered *Moringa oleifera* leaves.

RT (min)	MW (Da)	Chemical Formula	MS/MS (*m*/*z*)	Compound(s)	Amount (mg/100 g DW)
3.12	191	C_7_H_12_O_6_	191, 173, 127, 93, 85	Quinic acid	125.5 (3.104)
9.01	353	C_16_H_18_O_9_	191, 179, 135	3-Caffeoylquinic acid	101.3 (3.834)
12.96	353	C_16_H_18_O_9_	191, 179, 173, 135	4-Caffeoylquinic acid	36.2 (2.467)
14.51	593	C_27_H_30_O_15_	593, 473, 383, 353	Vicenin-2	26.2 (1.803)
18.35	354	C_16_H_18_O_9_	191	Chlorogenic Acid	40.3 (1.194)
20.42	609	C_27_H_30_O_16_	609, 301, 300	Quercetin-3-*O*-rutinoside	24.7 (1.608)
20.76	431	C_21_H_20_O_10_	341, 311, 283	Apigenin 8-*C*-glucoside	48.6 (1.005)
21.81	463	C_21_H_20_O_12_	463, 301, 300	Quercetin 3-*O*-glucoside	70.1 (3.824)
23.28	593	C_27_H_30_O_15_	593, 285	Kaempferol 3-*O*-rutinoside	50.6 (2.303)
24.68	447	C_21_H_20_O_11_	447, 285, 284, 255	Kaempferol 3-*O*-glucoside	33.1 (1.179)
25.37	477	C_22_H_22_O_12_	477, 315, 314, 243	Isorhamnetin 3-*O*-glucoside	69.3 (2.361)
27.89	283	C_15_H_10_O_5_		Apigenin	12.4 (0.906)
28.12	286	C_15_H_10_O_6_		Kaempferol	11.3 (0.731)
26.52	301	C_15_H_10_O_7_		Quercetin	9.3 (0.666)

Results are expressed as mg per 100 g of dry weight ± SD for each compound. Abbreviations: RT, retention times; MW, molecular weights.

**Table 2:** Bioactive compounds and antioxidant properties of powdered *Moringa oleifera* leaves.

**TPC**	2.86 ± 0.25	g GAE per 100 g of DW
**TCC**	1.64 ± 0.14	mg BCE per 100 g of DW
**Cha**	1.01 ± 0.04	g Ch per 100 g of DW
**Chb**	0.34 ± 0.02	g Ch per 100 g of DW
**DPPH** ^•^	5.67 ± 0.49	mmol TE per 100 g of FW
**ABTS** ^•+^	8.06 ± 0.57	mmol TE per 100 g of DW
**FRAP**	15.71 ± 2.19	mmol TE per 100 g of DW
**CAA_50_**	5.42 ± 0.43	µg of DW per mL cell medium

Values are expressed as mean ± SD of three experiments carried out in triplicate. Abbreviations: TPC, total polyphenol content; TCC, total carotenoid content; Cha, chlorophyll *a* content; Chb, chlorophyll *b* content; FRAP, ferric reducing antioxidant power; DPPH^•^, 2,2-diphenyl-1-picrylhydrazyl; ABTS^•+^, 2,2′-azino-bis(3-ethylbenzothiazoline-6-sulfonic acid); CAA, cellular antioxidant activity; GAE, gallic acid equivalents; BCE, β-carotene equivalent; TE, Trolox equivalent t.

**Table 3:** Antiproliferative activity of powdered *Moringa oleifera* leaves extract against HepG2, Caco-2, and MCF-7 tumour cell lines expressed as GI_50_ values (µg of dry weight per mL of cell medium).

**HepG2**	237.75 ± 4.37 ^c^
**Caco-2**	182.36 ± 4.52 ^d^
**MCF-7**	121.03 ± 3.71 ^a^

Values represented as mean ± SE of three experiments carried out in quadruplicate. Different lowercase letters indicate significant difference at *p* ≤ 0.05 as measured by Tukey’s multiple test.

## Data Availability

All data included in this study are available upon request by contacting the corresponding author.
